# Evaluation of muscular changes by ultrasound Nakagami imaging in Duchenne muscular dystrophy

**DOI:** 10.1038/s41598-017-04131-8

**Published:** 2017-06-30

**Authors:** Wen-Chin Weng, Po-Hsiang Tsui, Chia-Wei Lin, Chun-Hao Lu, Chun-Yen Lin, Jeng-Yi Shieh, Frank Leigh Lu, Ting-Wei Ee, Kuan-Wen Wu, Wang-Tso Lee

**Affiliations:** 1 Department of Pediatrics, National Taiwan University Hospital, Taipei, Taiwan; 2 Department of Pediatrics, College of Medicine, National Taiwan University, Taipei, Taiwan; 3 Department of Pediatric Neurology, National Taiwan University Children’s Hospital, Taipei, Taiwan; 4 Department of Medical Imaging and Radiological Sciences, College of Medicine, Chang Gung University, Taoyuan, Taiwan; 5 Institute for Radiological Research, Chang Gung University and Chang Gung Memorial Hospital at Linkou, Taoyuan, Taiwan; 6 Department of Medical Imaging and Intervention, Chang Gung Memorial Hospital at Linkou, Taoyuan, Taiwan; 7 Department of Physical Medicine and Rehabilitation, National Taiwan University Hospital Hsin-Chu Branch, Hsin-Chu, Taiwan; 8 Department of Pediatrics, Yong-He Cardinal Tien Hospital, Taipei, Taiwan; 9 Department of Physical Medicine and Rehabilitation, National Taiwan University Hospital, Taipei, Taiwan; 10 Department of Orthopaedic Surgery, National Taiwan University Hospital, Taipei, Taiwan; 11 Graduate Institute of Brain and Mind Sciences, National Taiwan University, Taipei, Taiwan

## Abstract

Duchenne muscular dystrophy (DMD) is the most common debilitating muscular disorder. Developing a noninvasive measure for monitoring the progression of this disease is critical. The present study tested the effectiveness of using ultrasound Nakagami imaging to evaluate the severity of the dystrophic process. A total of 47 participants (40 with DMD and 7 healthy controls) were recruited. Patients were classified into stage 1 (presymptomatic and ambulatory), stage 2 (early nonambulatory), and stage 3 (late nonambulatory). All participants underwent ultrasound examinations on the rectus femoris, tibialis anterior, and gastrocnemius. The results revealed that the ultrasound Nakagami parameter correlated positively with functional severity in the patients with DMD. The median Nakagami parameter of the gastrocnemius muscle increased from 0.50 to 0.85, corresponding to the largest dynamic range between normal and stage 3. The accuracy, sensitivity, and specificity of diagnosing walking function were 85.52%, 76.31%, and 94.73%, respectively. The Nakagami parameter of the rectus femoris and gastrocnemius muscles correlated negatively with the 6-minute walking distance in the ambulatory patients. Therefore, changes in the Nakagami parameter for the gastrocnemius muscle are suitable for monitoring disease progression in ambulatory patients and for predicting ambulation loss. Ultrasound Nakagami imaging shows potential for evaluating patients with DMD.

## Introduction

Duchenne muscular dystrophy (DMD) is the most common debilitating muscular disorder, affecting one in 3500 boys^1^. The characteristic features of this disease include progressive muscle weakness manifesting as delayed motor milestones and the classic Gowers’ sign. DMD is caused by mutation in the X-linked dystrophin gene, which causes a deficit of the dystrophin protein in muscle cells^2^, resulting in necrosis and regeneration accompanied by progressive replacement of muscles with fat and fibrotic tissues. When DMD is left untreated, ambulation loss typically occurs by the age of 9–14 years^3^, and death often occurs by the age of 20 years as a result of respiratory or cardiac complications^4^.

Standardized multidisciplinary care for DMD has been proposed in recent years, including the use of corticosteroids^4, 5^. Furthermore, several potential therapeutic approaches for patients with DMD are currently under investigation, including exon-skipping strategies^6^, stop codon readthrough^7^, gene repair therapy^8^, myostatin inhibition^9^, and antifibrotic agents^10^. However, coinciding with these advances is the need to develop appropriate and noninvasive measures for monitoring disease progression and evaluating the efficacy of potential therapies in clinical trials. Currently, the commonly used measures for assessing the clinical outcomes of DMD are functional rating scales including the 6-minute walk test (6MWT) and the North Star Ambulatory Assessment (NSAA)^11, 12^. Although these measures are beneficial, they are limited by patients’ effort and mood and can be used only in ambulatory boys.

Ultrasound and magnetic resonance imaging (MRI) of the muscles have been used to assess patients with neuromuscular disorders including DMD^13, 14^. Ultrasound offers some benefits over MRI because it is inexpensive, time saving, and can be performed whenever needed in outpatient clinics. In general, the brightness of an ultrasound B-mode image is determined by the amplitude of backscattered signals, which are formed from the summation of echoes contributed by each scatterer in a tissue. Recent reports have demonstrated the potential of quantitative muscle ultrasound for evaluating DMD on the basis of the amplitude of backscattered signals^15–17^. In boys with DMD, an increase in intramuscular fat and connective tissues augments the echo intensity in ultrasound imaging^13, 18^, and this becomes exacerbated with disease progression and a reduction in strength and function over time^16, 17^. Although analyzing the backscatter intensity is useful for characterizing DMD, its physical meaning corresponds to the echogenicity of scatterers, but not to information associated with scattering structures, which may be beneficial for interpreting muscle pathology.

Recently, the technique of acoustic structure quantification (ASQ) has gained attention as a tool for characterizing tissues^19–21^. The basic concept of ASQ is to analyze the statistical distribution of raw backscattered signals (i.e., echo amplitude distribution) for ultrasound tissue characterization. Specifically, different scattering structures result in different backscattered statistics; therefore, modeling the backscattered statistics is an effective approach for characterizing scatterer properties^22^. The homodyned K distribution has been suggested as a general model for describing backscattered statistics^22, 23^. Considering the analytical complexity of the homodyned K distribution, the Nakagami distribution provides a useful approximation for ultrasound backscattering and has been the most frequently adopted model for tissue characterization because of its simplicity and low computational complexity^22^. Ultrasound Nakagami imaging based on a Nakagami parametric map visualizes changes in the echo amplitude distribution and has already been used in various applications such as breast tumor classification^24^ and liver fibrosis detection^25–28^. In the present study, we analyzed the ultrasound backscattered statistics of muscle ultrasound data to investigate whether Nakagami parametric imaging reflects the severity of the dystrophic process in patients with DMD.

## Materials and Methods

### Study Population

This study was approved by the Institutional Review Board of National Taiwan University Hospital. All participants signed informed consent forms. All experimental methods were conducted in accordance with the approved guidelines. Between April 2015 and June 2016, 40 patients were recruited in the joint clinics of neuromuscular disorders in Department of Pediatrics, National Taiwan University Hospital. All patients had clinical presentations consistent with DMD and received their diagnosis according to a muscle biopsy with absent dystrophin and/or genetic confirmation of DMD. Demographic data on the patients were collected from the Department of Pediatrics, National Taiwan University Hospital. After referring to a review report^4^, the severity of DMD for each patient was classified into three stages: stage 1 (presymptomatic, early ambulatory, and late ambulatory), stage 2 (early nonambulatory), and stage 3 (late nonambulatory). Seven children (age, 3–16 years) without history of weakness or neuromuscular disorders were also recruited as controls.

### Ultrasound Examination

A portable clinical ultrasound scanner (Model 3000, Terason, Burlington, MA, USA) and linear transducer with a central frequency of 7 MHz, 128 elements, and a pulse length of approximately 0.7 mm (Model 12L5A, Terason) were used for sonographic imaging. All participants underwent a standard-care ultrasound examination on three muscles: the rectus femoris, tibialis anterior, and gastrocnemius. For each muscle, a skilled physician obtained three valid grayscale images (no acoustic shadowing artifacts and exclusion of large vessels in the region of analysis) using the sagittal scanning approach. The imaging focus and depth were set at 2 and 4 cm, respectively. The grayscale image data were stored in ULT file, which is dedicated to the Terason ultrasound system. Raw image data consisting of 128 backscattered radio-frequency (RF) signals (i.e., scan lines) recorded at a sampling rate of 30 MHz corresponding to each grayscale image were obtained by converting the ULT files into MAT files, which were read using the MATLAB for offline data processing and ultrasound Nakagami imaging.

### 6MWT

In addition to the ultrasound examination, all ambulatory boys with DMD who were older than 5 years (n = 16) completed the 6MWT in accordance with the American Thoracic Society guidelines^29^.

### Ultrasound Nakagami imaging algorithm

The backscattered statistics of ultrasound echoes measured from biological tissues can be classified as pre-Rayleigh, Rayleigh, and post-Rayleigh distributions^30^. The Nakagami distribution fits well with these three distributions. The probability distribution function of the backscattered envelope *r* under the Nakagami model is given by^31^

1 $$f(r)=\frac{2{m}^{m}{r}^{2m-1}}{{\rm{\Gamma }}(m){{\rm{\Omega }}}^{m}}\exp (-\frac{m}{{\rm{\Omega }}}{r}^{2})U(r),$$

where Γ(∙) and *U*(∙) are the gamma and unit step functions, respectively. Let *E*(∙) denote the statistical mean. The scaling parameter Ω and the Nakagami parameter *m* associated with the Nakagami distribution can be respectively obtained using

2 $$\Omega =E({R}^{2})$$

and

3 $$m=\frac{{[E({R}^{2})]}^{2}}{E[{R}^{4}]-{[E({R}^{2})]}^{2}}.$$

where *R* is the envelope (i.e., amplitude) of the ultrasound signals. The Nakagami parameter is the shape parameter of the Nakagami distribution, the value of which is interpreted as follows: (i) *m* < 0.5 is Nakagami-gamma distribution (few scatterers with gamma-distributed scattering cross-sections in the resolution cell), (ii) 0.5 ≤ *m* ≤ 1 is a pre-Rayleigh distribution (few scatterers or strong scatterers mixed with randomly distributed scatterers in the resolution cell), (iii) *m* = 1 is a Rayleigh distribution (a large number of randomly distributed scatterers in the resolution cell), and (iv) *m* > 1 is a post-Rayleigh distribution (periodically located scatterers and randomly distributed scatterers in the resolution cell). Therefore, the Nakagami parameter enables the quantification of echo amplitude distributions with specific physical meanings associated with scattering structures.

To obtain high-quality images and visualization through Nakagami imaging, the window-modulated compounding (WMC) technique was used in this study. Details on the compounding Nakagami imaging algorithm based on the sliding window technique are described in a recent study^32^. A Nakagami image *M*_N_ (*x*, *y*) constructed using the window side lengths corresponding to *N* times the pulse length of the transducer can be formulated on the basis of the convolution operator, as follows:

4 $${M}_{N}(x,y)=\frac{{[{\hat{R}}^{\mathop{2}\limits^{\circ }}\otimes {W}_{N}]}^{\mathop{2}\limits^{\circ }}}{{\hat{R}}^{\mathop{4}\limits^{\circ }}\otimes {W}_{N}-{[{\hat{R}}^{\mathop{2}\limits^{\circ }}\otimes {W}_{N}]}^{\mathop{2}\limits^{\circ }}}.$$

where $\hat{R}$ is an envelope image, *W*_N_ is a sliding window with side lengths of *N* times the pulse length, and ${\hat{R}}^{\mathop{{\rm{n}}}\limits^{\circ }}$ is the *n*th power of the Hadamard product of $\hat{R}$. In the WMC technique, the Nakagami parameter is obtained by calculating the average of all Nakagami images derived using various window sizes, as follows:

5 $${M}_{WMC}(x,y)=\frac{1}{N}\sum _{i=1}^{N}{M}_{i}(x,y).$$

where *N* represents the number of frames used for Nakagami image compounding.

### Data analysis

Raw image data were employed to obtain the envelope image by using the absolute value of the Hilbert Transform of each backscattered RF signal. B-mode images were formed using log-compressed envelope images in a dynamic range of 40 dB. The uncompressed envelope image was applied in equation (4) to construct Nakagami images (*N* = 1–7) for compounding with equation (5). In this study, *N* was set to 7 to reduce the estimation error to < 5% compared with that of the conventional Nakagami image^33^. For each image, a primary region of interest (ROI) was manually set by the physician to calculate the mean of the pixel values (i.e., the Nakagami parameter) within the ROI, which served as a biomarker of muscle tissues.

### Statistical analysis

The Nakagami parameter as a function of the DMD stage is expressed as the median and interquartile range (IQR). One-way ANOVA was performed to compare the difference in the Nakagami parameter between groups (*p* < 0.05 was considered statistically significant). Receiver operating characteristic (ROC) curve analysis with 95% confidence intervals (CIs) was performed to obtain the area under the ROC curve (AUROC), which was used to determine the predictive value of the Nakagami parameter for diagnosing the walking function: stage 1 (ambulatory) versus stages 2 and 3 (nonambulatory). The sensitivity, specificity, and accuracy were then calculated. The measurement values obtained from the 6MWT (reported in meters) and Nakagami imaging were compared to calculate the correlation coefficient *r* by using the linear curve fitting model y = y0 + ax. All statistical analyses were performed using SigmaPlot Version 12.0 (Systat Software, Inc., CA, USA).

## Results

In total, 40 patients with DMD (age, 2–24 years) and seven controls (age, 3–16 years) were included in the study. Of the 40 patients, 21 (age, 7.62 ± 2.13 years) were in stage 1, 11 (age, 12.1 ± 1.80 years) were in stage 2, and eight (age, 17.38 ± 3.16 years) were in stage 3. The participants’ demographic data, including their clinical symptoms, disease stage, and age, are summarized in Table 1.

**Table 1:** The demographic data and DMD stage definitions.

Stage	Clinical symptoms	Descriptions	Age (years) (range)	Numbers of participants
Control	No weakness	Volunteers without neuromuscular disorders or weakness	8 ± 4 (3–16)	7
Stage 1	Presymptomatic	Can be diagnosed at this stage if creatine kinase found to be raised or if positive family history	7.62 ± 2.13 (2–11)	21
		Might show developmental delay but no gait disturbance		
	Early ambulatory	Gowers’ sign		
		Waddling gait		
		Might be toe walking		
		Can climb stairs		
	Late ambulatory	Increasingly laboured gait		
		Losing ability to climb stairs and rise from floor		
Stage 2	Early non-ambulatory	Might be able to self propel for some time	12.1 ± 1.80 (9–14)	11
		Able to maintain posture		
		Might develop scoliosis		
Stage 3	Late non-ambulatory	Upper limb function and postural maintenance is increasingly limited	17.38 ± 3.16 (15–24)	8

**Figure 1:**
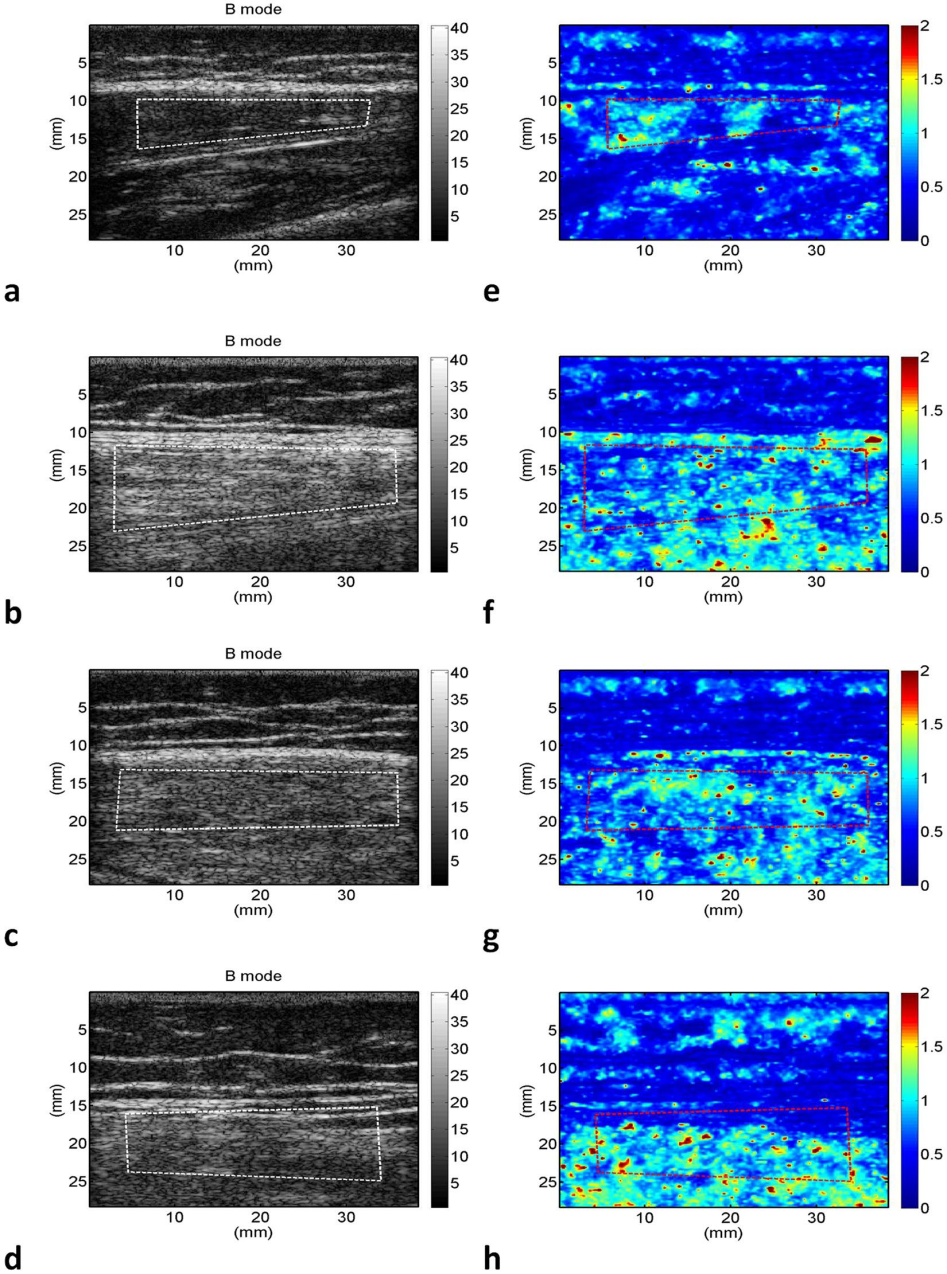
Grayscale B-mode images of the rectus femoris muscle of the (**a**) controls; (**b**) stage 1 DMD patients; (**c**) stage 2 DMD patients; and (**d**) stage 3 DMD patients. (**e**–**h**) Corresponding WMC Nakagami images showing an increase in the brightness of the WMC Nakagami image with an increase in the DMD stage.

**Figure 2:**
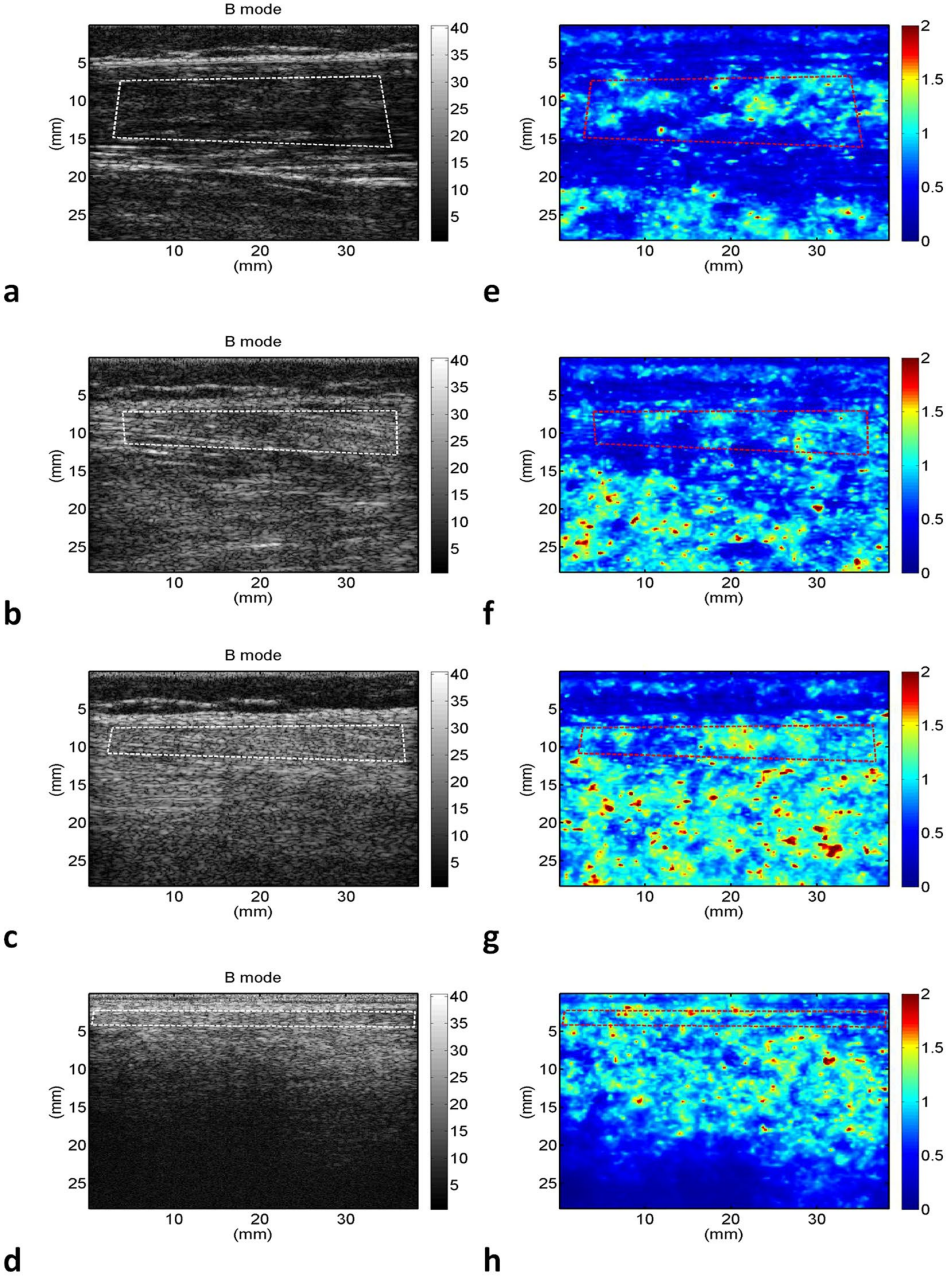
Grayscale B-mode images of the tibialis anterior muscle of the (**a**) controls; (**b**) stage 1 DMD patients; (**c**) stage 2 DMD patients; and (**d**) stage 3 DMD patients. (**e**–**h**) Corresponding WMC Nakagami images showing an increase in brightness of the WMC Nakagami image with an increase in the DMD stage, representing an increase in the Nakagami parameter.

**Figure 3:**
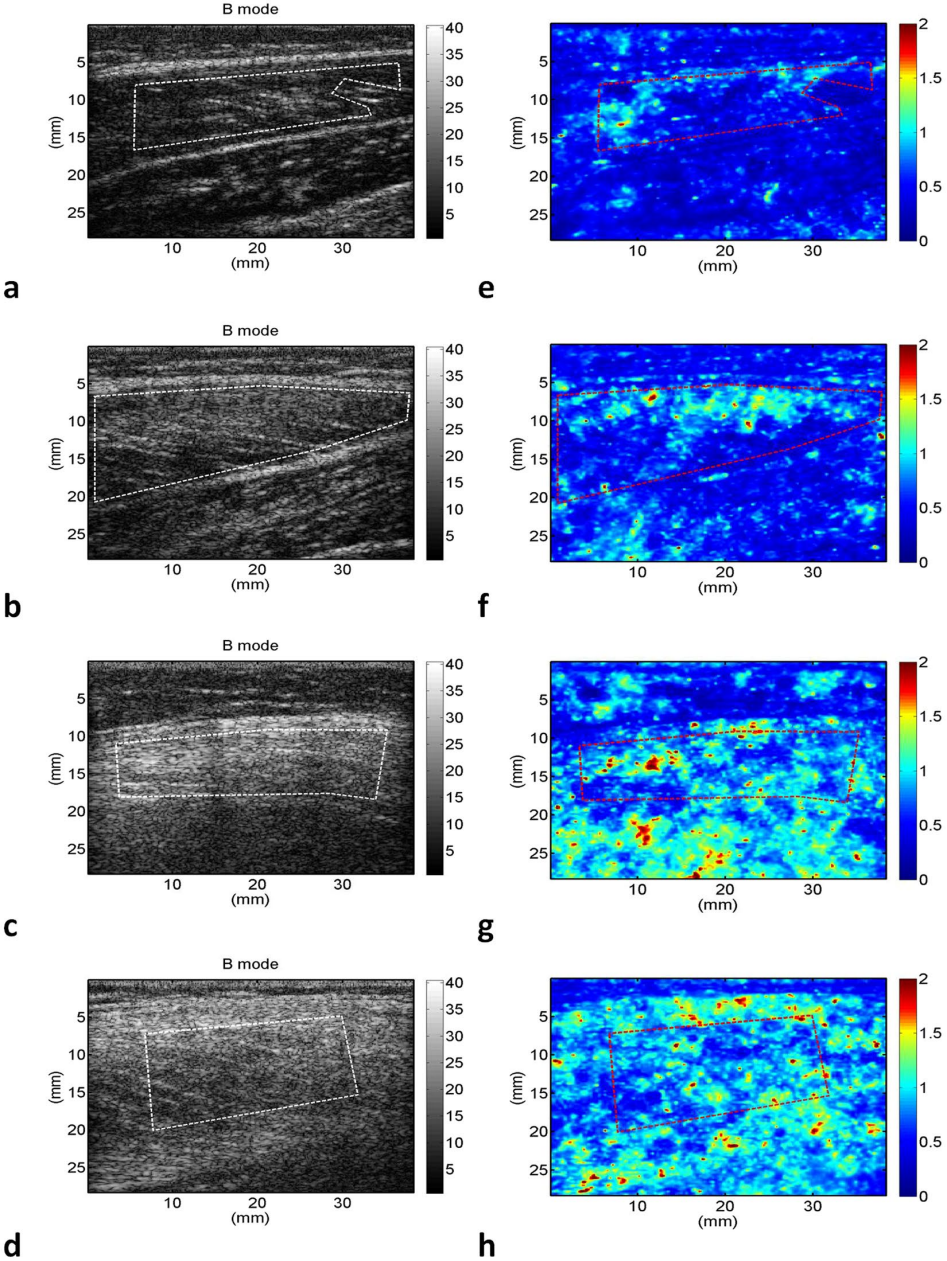
Grayscale B-mode images of the gastrocnemius muscle of the (**a**) controls; (**b**) stage 1 DMD patients; (**c**) stage 2 DMD patients; and (**d**) stage 3 DMD patients. (**e**–**h**) Corresponding WMC Nakagami images showing an increase in the brightness of the WMC Nakagami image from control to stage 3.

**Figure 4:**
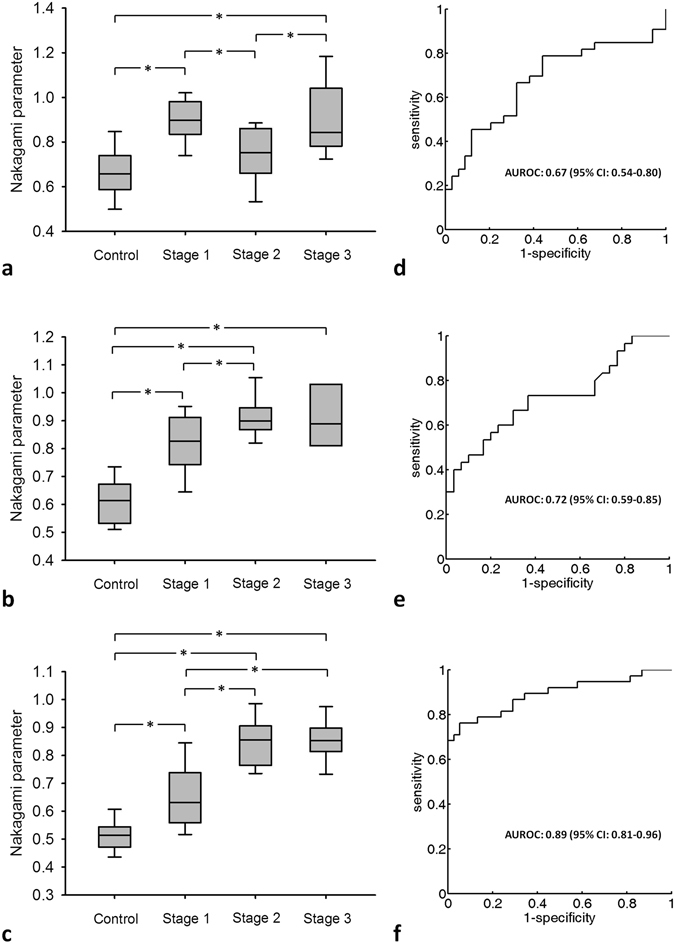
Nakagami parameters corresponding to different DMD stages. Data are expressed using box plots. The Nakagami parameter increases with the DMD stage, indicating that the backscattered statistics gradually change from a pre-Rayleigh distribution to a Rayleigh distribution. (**a**) Rectus femoris; (**b**) tibialis anterior; (**c**) gastrocnemius. (**d**–**f**) Corresponding ROC curves. The AUROC for the Nakagami parameter of the gastrocnemius muscle (0.89) is higher than those of the rectus femoris and tibialis anterior muscles.

The grayscale B-mode and WMC Nakagami images of the rectus femoris (Fig. 1), tibialis anterior (Fig. 2), and gastrocnemius (Fig. 3) muscles were obtained from the controls and patients with DMD. For each muscle, the grayscale B-mode image showed no prominent change in muscle echogenicity from stage 1 to stage 3. By contrast, the brightness of the WMC Nakagami image tended to increase with age and the DMD stage, representing an increase in the Nakagami parameter (Fig. 4a–c). However, the Nakagami parameter in the controls did not increase with age (data not shown). The Nakagami parameter of all the muscles differed significantly between the patients with DMD and controls (matched by age) (Fig. 4). Increases were observed in the median Nakagami parameter for the rectus femoris muscle, from 0.72 (IQR: 0.64–0.79) to 0.84 (IQR: 0.78–1.03; Fig. 4a); for the tibialis anterior muscle, from 0.64 (IQR: 0.62–0.70) to 0.88 (IQR: 0.81–0.95); and for the gastrocnemius muscle, from 0.50 (IQR: 0.46–0.56) to 0.85 (IQR: 0.81–0.88) (Fig. 4b and c). A significant decrease in the Nakagami parameter was detected in the rectus femoris muscle from stage 1 to stage 2, followed by a significant increase in the Nakagami parameter from stage 2 to stage 3 (Fig. 4a). By contrast, the Nakagami parameters obtained for the tibialis anterior and gastrocnemius muscles persistently increased from control to stage 2.

Among the dynamic ranges of the Nakagami parameters (i.e., differences between the maximum and minimum values) obtained for the rectus femoris, tibialis anterior, and gastrocnemius muscles, that for the gastrocnemius muscle was the highest (0.35–1.04). Higher dynamic ranges of this parameter may improve the diagnostic performance of staging DMD (Fig. 4d–f). The cutoff values of the Nakagami parameter, which was measured for the rectus femoris, tibialis anterior, and gastrocnemius muscles in order to differentiate the ambulatory and nonambulatory patients, were 0.85, 0.87, and 0.73, respectively. The AUROCs (95% CIs) were 0.67 (0.54–0.80), 0.72 (0.59–0.85), and 0.89 (0.81–0.96) for the WMC Nakagami imaging of the rectus femoris, tibialis anterior, and gastrocnemius muscles, respectively. These results indicated that the Nakagami parameter obtained from the gastrocnemius muscle was more sensitive for detecting dystrophic progression in ambulatory patients with DMD. The accuracy, sensitivity, and specificity of using the gastrocnemius as a target muscle for measurements were 85.52%, 76.31%, and 94.73%, respectively. The results for differentiating between the ambulatory and nonambulatory patients are summarized in Table 2. The AUROC measured from the gastrocnemius muscle was higher than those measured from the rectus femoris and tibialis anterior muscles, indicating improved performance in DMD evaluation.

**Table 2:** Clinical performance of ultrasound Nakagami imaging in diagnosing walking function.

Muscle		Rectus femoris	tibialis anterior	Gastrocnemius
Median (IQR) of the Nakagami parameter	Control	0.72 (0.64–0.79)	0.64 (0.62–0.70)	0.50 (0.46–0.56)
	Stage 1	0.88 (0.83–0.97)	0.82 (0.75–0.90)	0.63 (0.55–0.73)
	Stage 2	0.75 (0.67–0.85)	0.89 (0.86–0.93)	0.85 (0.77–0.90)
	Stage 3	0.84 (0.78–1.03)	0.88 (0.81–0.95)	0.85 (0.81–0.88)
Dynamic range of the parameter		0.48–1.22	0.51–1.09	0.35–1.04
Cutoff value		0.85	0.87	0.73
Sensitivity, %		66.6	66.66	76.31
Specificity, %		67.6	70	94.73
Accuracy, %		67.1	68.33	85.52
LR+		2.06	2.22	14.5
LR−		0.49	0.47	0.25
PPV, %		66.66	68.96	93.54
NPV, %		67.64	67.74	80
AUROC		0.67	0.72	0.89
(95% CI)		(0.54–0.80)	(0.59–0.85)	(0.81–0.96)

LR+: positive likelihood ratio, LR−: negative likelihood ratio, PPV: positive predictive value, NPV: negative predictive value, AUROC: area under the receiver operating characteristics curve.

**Figure 5:**
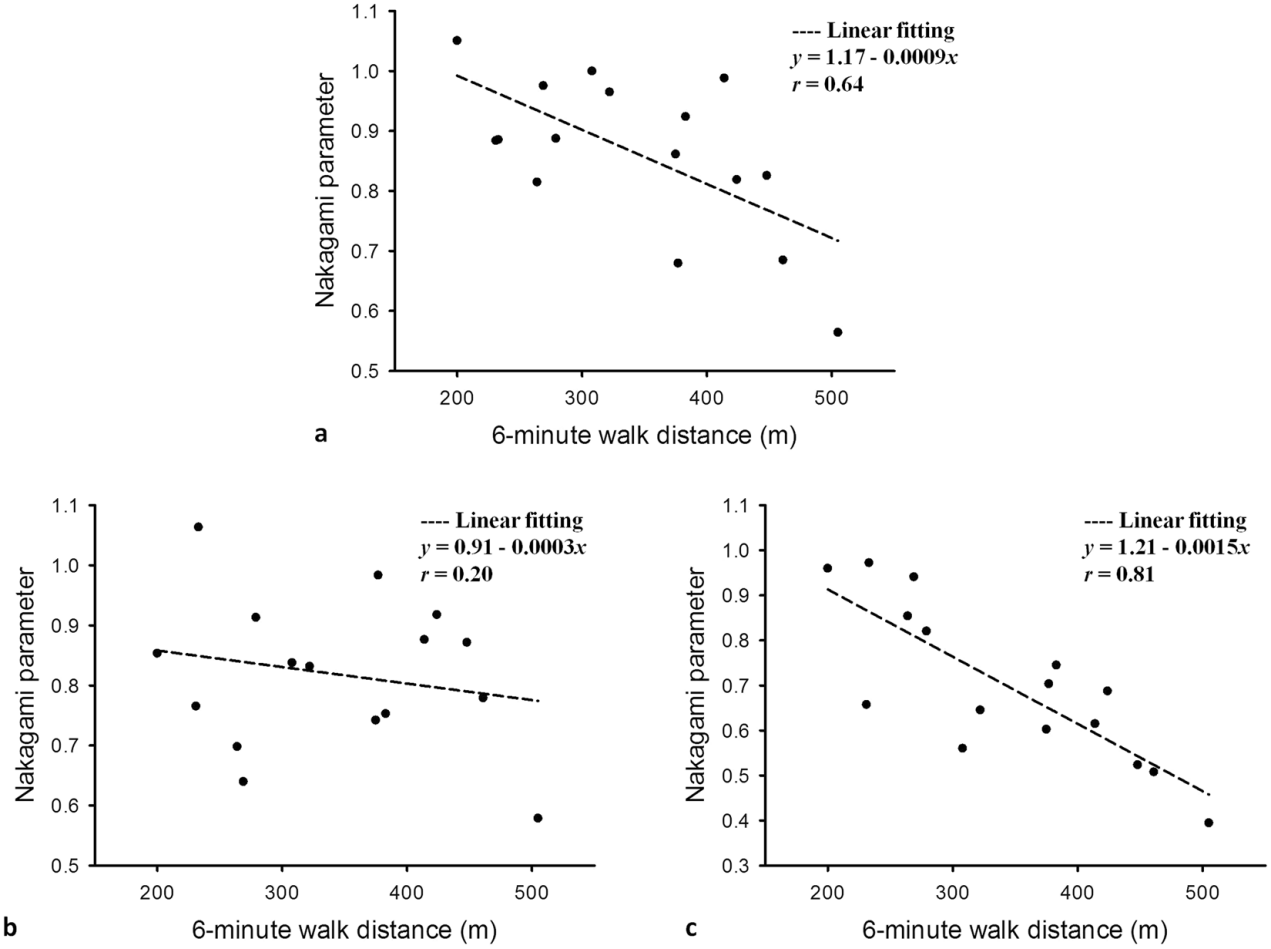
Correlations between the Nakagami parameters and 6MWT results in ambulatory patients with DMD (age >5 years). Data are expressed as the correlation coefficient *r* and were obtained using a linear curve fitting model y = y0 + ax. The Nakagami parameters in the rectus femoris and gastrocnemius muscles exhibited an increase with shorter walking distances. (**a**) Rectus femoris (*r* = 0.64); (**b**) tibialis anterior; and (**c**) gastrocnemius (*r* = 0.81).

Subsequently, we compared the results of 6MWT with the Nakagami parameters corresponding to the rectus femoris, tibialis anterior, and gastrocnemius muscles, respectively (Fig. 5). Overall, the Nakagami parameter increased with shorter walking distances. In particular, relatively significant correlations were obtained for the rectus femoris (*r* = 0.64) and gastrocnemius (*r* = 0.81) muscles, which are primarily responsible for the ambulatory status.

## Discussion

Recent advances in several potential therapeutic strategies for DMD have led to greater need for appropriate and noninvasive measures for evaluating disease severity, which correlates with pathological changes. In the present study, we evaluated whether muscle ultrasound using Nakagami parametric imaging can indicate the severity of the dystrophic process in patients with DMD by analyzing the ultrasound backscattered statistics. This is the first study to demonstrate that the ultrasound Nakagami parameter increases with functional severity in patients with DMD.

With the advantages of real-time and dynamic assessment, muscle ultrasound is a very useful tool in the diagnosis and longitudinal follow-up of DMD, because this disorder results in muscle destruction with progressively increasing fibrosis and fatty infiltration. Compared with normal muscles, which exhibit low echo intensity, dystrophic muscles are characterized by a homogenous increase in echo intensity resulting from increased intramuscular fat and fibrosis^18, 34^. Recent reports have shown that in evaluating DMD, quantitative muscle ultrasound based on backscatter intensity analysis yields reliable correlations with disease severity^15–17^. Although backscatter intensity analysis is useful for characterizing DMD, it is not directly informative for different scattering structures that may be more beneficial for interpreting muscle pathology. The Nakagami parameter can differentiate different scatterer concentrations and is less affected by signal intensity^35^. In the present study, Nakagami images measured from three different muscles of the lower extremities showed a similar increasing trend in the Nakagami parameters with progression in the DMD stage. In all the muscles investigated in this study, we observed a significant difference in the Nakagami parameter between the patients with DMD and age-matched healthy participants, suggesting that Nakagami imaging is a sensitive and objective technique for determining muscle pathology in dystrophinopathies.

The Nakagami parameter is a shape parameter and follows a Rayleigh distribution according to different scatterer concentrations. In Nakagami imaging, the parameter moves from a pre-Rayleigh to a Rayleigh distribution if the tissue distribution becomes more homogenous (and vice versa)^33^. Our study results reveal a decrease in the Nakagami parameter in the rectus femoris muscle after loss of free ambulation (from stage 1 to stage 2), and then an increase in the Nakagami parameter from stage 2 to stage 3. Because the ambulatory status of patients with DMD depends largely on the involvement of the rectus femoris muscle, the initial increase in the Nakagami parameter can be explained by homogenously increased fibrosis following muscle cell necrosis and inflammation during the ambulatory period^36^, whereas the decrease in the Nakagami parameter after the loss of free ambulation represents fatty infiltration following disuse atrophy, which has also been observed in a prior muscle MRI study^37, 38^. Using the B-mode intensity in the clinical evaluation of different muscle characterizations might have some disadvantages. First, to more clearly visualize the change in muscles, a typical ultrasonic scanner allows operators to adjust different system parameters such as the system gain, time-gain compensation, and dynamic range. Moreover, different ultrasonic scanners produced by different manufacturers have different procedures for signal and image processing. Therefore, the B-mode image is easily affected by system factors. Second, the specular reflection is highly angle-dependent. When the sound beam is perpendicular to the tissue interface, the transducer can receive a considerable amount of reflected acoustic energy. Otherwise, less energy is received. This implies that the brightness of B-mode images also relies on the skill and training of the operator. The proposed method, ultrasound Nakagami imaging, constructs images from unprocessed backscattered envelope signals and thus may reduce the system effects on results and provide a more objective evaluation of DMD. Our results further support that Nakagami imaging is more sensitive than quantitative ultrasound intensity analysis in detecting subtle changes in homogeneity and different tissue characterizations.

The optimal muscle groups for evaluating dystrophic changes over time in DMD may differ depending on the patient age and disease severity^16^. In our study, we observed no obvious changes in the Nakagami parameter for the distal lower extremities, including the tibialis anterior and gastrocnemius muscles, after the loss of ambulation. This implies that the lower extremities may not be an ideal muscle group for examining age-related changes with ultrasound in wheelchair-dependent patients. Previous studies performing quantitative ultrasound intensity analyses have shown similar findings regarding the muscle echo intensity of the lower extremities increasing over time being more sensitive to changes in younger boys, whereas the that of the upper extremities has been shown to be more sensitive to changes in boys older than 8 years^16^. Additional studies of the Nakagami images of upper extremities are warranted to clarify the pattern of muscle involvement with age in patients with DMD, and to select the optimal muscle for monitoring disease progression in wheelchair-dependent boys.

In our study, the gastrocnemius muscle exhibited the largest dynamic range in the Nakagami parameter during dystrophic progression. Higher dynamic ranges may improve the evaluation and discrimination of staging DMD. Because the gastrocnemius muscle is more sensitive and responsive to progressive changes in the muscle architecture in ambulatory boys with DMD, changes in the Nakagami parameter for the gastrocnemius muscle are more suitable for monitoring disease progression in ambulatory patients. A previous study of muscle MRI in patients with DMD also suggested that the gastrocnemius muscle was the earliest and most severely affected and could be useful for disease monitoring in ambulatory boys^14^. Muscle ultrasound offers some benefits over muscle MRI because it is inexpensive, time saving, and can be performed whenever needed in outpatient clinics. Moreover, the statistics of the Nakagami parameter for the gastrocnemius muscle also show the highest AUROC for predicting the time of loss of ambulation in DMD, with a sensitivity of 76.31% and specificity of 94.73%.

Recent advances in potentially effective therapeutic approaches for patients with DMD have stimulated the need for outcome measures to be developed through clinical trials. The primary ambulatory outcome measures in recent clinical trials have been the 6MWT and the NSAA^11, 12^. In the present study, we found that the Nakagami parameter of the rectus femoris and gastrocnemius muscles, which are primarily responsible for the ambulatory status, correlated inversely with the 6WMT results in ambulatory patients. Considering that these two functional rating scales are limited by the patients’ effort and mood and can only be used in older and ambulatory boys, the Nakagami parameter may provide a more objective and reliable measure of pathological changes in muscles. Furthermore, Nakagami ultrasound can be applied easily in younger or nonambulatory patients with DMD. Further investigation is needed to verify whether the Nakagami parameter is adequately sensitive for detecting the early dystrophic process in very young patients.

This study encountered some limitations. It was performed using a cross-sectional design, and the correlation of Nakagami parameter changes with disease progression and disease severity was not fully explored. Further longitudinal investigations are needed to establish whether Nakagami parameters provide a reliable correlation with disease severity and progression in DMD.

## Conclusion

To the best of our knowledge, this is the first pilot study to evaluate dystrophic muscles using ultrasonic Nakagami imaging in patients with DMD. The results demonstrated that ultrasound Nakagami parameters increase with functional severity in patients with DMD, suggesting that Nakagami imaging is a sensitive and objective technique for assessing DMD severity. Nakagami imaging is also more sensitive than quantitative ultrasound intensity analysis for detecting subtle changes in homogeneity and characterizing different tissues. From a therapeutic perspective, the application of Nakagami imaging in DMD may provide a bridge between research on the muscle architecture and function, and the technique may be suitable for inclusion in ongoing therapeutic trials.
